# Ultrasonographic evaluation of neck extensor muscle thickness in smartphone users

**DOI:** 10.1590/1806-9282.20242098

**Published:** 2025-07-07

**Authors:** Ezgi Akyildiz Tezcan, Ali Erbay

**Affiliations:** 1 Selcuk University, Faculty of Medicine, Department of Physical Medicine and Rehabilitation – Konya, Turkey.; 2 Cumra State Hospital, Department of Radiology – Konya, Turkey.

**Keywords:** Smartphone, Ultrasonography, Neck muscles, Neck pain, Posture

## Abstract

**OBJECTIVE:**

The aim of this study was to investigate the relationship between smartphone usage and neck extensor muscle thickness through ultrasonographic evaluation.

**METHODS:**

A prospective cross-sectional study was conducted with 75 participants aged 18–40 years. Demographic data, smartphone usage patterns, and neck extensor muscle thickness were assessed. Smartphone addiction was evaluated using the short version of the Smartphone Addiction Scale. Statistical analyses included appropriate tests, with p<0.005 being considered statistically significant.

**RESULTS:**

Participants with smartphone addiction demonstrated statistically significantly higher Neck Disability Index scores compared to their non-addicted counterparts (p=0.001). Although no statistically significant differences were observed in muscle thickness between the addicted and non-addicted groups, smartphone usage duration negatively correlated with dominant trapezius thickness (**ρ**=-0.362, p=0.028). The age of smartphone use onset was positively correlated with the thickness of several muscles, including the splenius capitis on both the dominant (**ρ**=0.358, p=0.030) and non-dominant sides (**ρ**=0.487, p=0.002), and the average thickness of the splenius capitis (**ρ**=0.442, p=0.006) and semispinalis cervicis muscles (r=0.349, p=0.034). Regression analysis confirmed significant relationships between the age of onset and neck extensor muscle thickness.

**CONCLUSIONS:**

Prolonged smartphone use, especially when initiated at an early age, is associated with changes in neck extensor muscle morphology. Ultrasonographic evaluation revealed correlations between usage patterns and muscle thickness, highlighting the importance of preventive measures and ergonomic interventions to mitigate potential musculoskeletal risks associated with excessive smartphone use.

## INTRODUCTION

The ubiquity and convenience of smartphones have led to their deep integration into everyday life, making them a cornerstone of contemporary society^1^. The exponential increase in smartphone usage is largely attributed to the substantial benefits they offer, such as enhanced comfort, connectivity, and accessibility^1^. Since their widespread availability in 2011, smartphones have transformed how we communicate and interact, profoundly influencing various aspects of human behavior and lifestyle^1,2^.

Even though smartphones offer numerous benefits, they also raise concerns about potential negative effects on our bodies, particularly on our musculoskeletal health^1-3^. With billions of users worldwide, identifying potential health risks and developing preventive strategies are crucial for public health. This study focuses on a specific aspect of this impact: the relationship between smartphone users and the thickness of neck extensor muscles. Neck extensor muscles play a crucial role in maintaining head posture and alignment, essential for various daily activities and overall spinal health. The advent of smartphones has led to a significant increase in the time individuals spend with their heads flexed forward, a posture that may ­influence the morphology and function of these muscles^4^. Although previous studies have examined the effects of smartphone use on neck disability^5^, cervical disc degeneration^6^, and neck extensor muscle endurance^7^, no morphological evaluation has been made regarding its effect on neck extensor muscles. This will give us enough motivation for the current study.

Ultrasonography is a non-invasive and dependable ­imaging technique, serves as an effective tool for measuring muscle thickness, and is extensively utilized in musculoskeletal research^8^. This study employs ultrasonographic methods to investigate the potential link between prolonged smartphone use and alterations in neck extensor muscle thickness. It aims to provide definitive evidence on how sustained smartphone engagement might influence these muscle characteristics.

The research will thoroughly explore this relationship by assessing various factors, including the age at which ­individuals start using smartphones, the duration and intensity of their use, potential smartphone addiction, and daily usage patterns. Gaining an understanding of this relationship will shed light on the wider consequences of mobile technology on physical health and inform strategies to counteract any negative impacts.

## METHODS

### Study design and participants

This prospective cross-sectional study was approved by the local ethics committee and was conducted in adherence to the Declaration of Helsinki (2000, Edinburgh). Written informed consent was obtained from all participants. Recruitment took place at the physical medicine and rehabilitation outpatient clinic between January and March 2024. Participants, aged 18–40 years, were identified during routine visits and were invited to join the study. The inclusion criteria required participants to be aged between 18 and 40 years, have regular use of a smartphone for at least 1 year, and be able and willing to provide written informed consent. The exclusion criteria included pregnancy; history of cervical spine surgery, trauma, or congenital anomalies; malignancy; major kidney/liver disease; uncontrolled diabetes; neurological disorders; and an inability to maintain a neutral head-and-neck position during ultrasonographic evaluation. Eligible participants were informed of the study's purpose, procedures, risks, and benefits. Demographic data and details on smartphone use (age of onset, duration, and daily usage) were recorded. The participants were assured of their right to withdraw at any time, and the confidentiality of their information was maintained.

### Smartphone addiction

The Smartphone Addiction Scale-Short Version (SAS-SV) was used to assess the level of smartphone addiction among participants^9,10^. This instrument includes 10 items, rated on a Likert scale from 1 (strongly disagree) to 6 (strongly agree). A threshold score of 31 for males and 33 for females was used to identify smartphone addiction, based on the existing literature. Participants’ scores were used to categorize them into groups for comparative analysis.

### Ultrasound measurement

Ultrasound evaluations were conducted using a 3–13 MHz linear ultrasound probe (Mindray Medical International Limited, Shenzhen, China) by a radiologist with more than 20 years of experience, who was blinded to the participants’ group allocations. To maintain the validity and consistency of the measurements, a standardized protocol was followed for each examination. The C4 vertebral process was chosen as the standard anatomical landmark^10^. Participants were seated in a neutral head-and-neck position for the ultrasound ­examinations, during which the probe was placed transversely at the C4 level. Bilateral measurements at rest were taken of the thickness of the multifidus, semispinalis capitis, semispinalis cervicis, splenius capitis, and trapezius muscles, capturing the maximum distance between the fascial borders of each ­muscle. The average muscle thickness was then calculated from the bilateral data for both the dominant and non-dominant hand sides and was recorded.

### Statistical analysis

Statistical analyses were conducted using SPSS 21.0 software (IBM Corp., Chicago, IL, USA). Categorical data were presented as frequencies and percentages, while quantitative data were described as mean±standard deviation (SD) or medians with ranges, depending on the distribution. Normality was assessed using histograms, skewness, kurtosis, and Shapiro-Wilk test. For comparisons between two groups, the independent samples t-test was used for normally distributed variables and the Mann-Whitney U test for non-normally distributed ­variables. One-way analysis of variance (ANOVA) and Kruskal-Wallis H test were applied for multiple group comparisons. Analysis of covariance (ANCOVA) controlled for confounders, with log transformations for non-normal data. Levene's test was used to assess variance and homogeneity for grouped variables. Categorical variables were compared using Pearson's chi-square or Fisher's exact test. Relationships among variables were examined using ­simple linear regression, with log-transformed data and ­verification of linearity assumptions. Outliers were removed using Mahalanobis distance. Correlations were assessed with Pearson's or Spearman's coefficients. A type I error rate of 5% was used, with statistical significance set at p<0.05.

## RESULTS

A total of 75 individuals participated in the study, with the majority being women (68%). Demographic data, smartphone usage parameters, and neck extensor muscle thickness did not differ significantly between groups with and without smartphone addiction. However, the Neck Disability Index (NDI) was statistically significantly higher among individuals with smartphone addiction, with a median (min–max) of 11 (2–29) compared to 6 (0–21) in the non-addicted group (p=0.001). Detailed data are presented in Tables 1 and 2.

**Table 1:** Comparison of demographic information and smartphone usage parameters in patients with and without smartphone addiction.

Parameters	Smartphone addicted (n=37, 49.3%)	Non-addicted (n=38, 50.7%)	p-value
Age; year, mean (SD)	29.73 (6.85)	31.47 (4.48)	0.198
Height; cm, median (min–max)	167 (152–183)	163.5 (155–186)	0.370
Weight; kg, median (min–max)	70.89 (14.62)	68.97 (14.38)	0.569
BMI; kg/cm^2^, mean (SD)	25.02 (3.67)	24.82 (3.75)	0.822
Gender; female; n (%)	24 (64.86%)	27 (71.05%)	0.744
Smoking habit; smoker, n (%)	12 (32.43%)	16 (42.10%)	0.531
Hand dominancy; right, n (%)	35 (94.59%)	33 (86.84%)	0.430
Active sports more than 2 days/week; positive, n (%)	12 (32.43%)	11 (28.95%)	0.939
Active job, n (%)	12 (32.43%)	19 (50%)	0.190
Neck Disability Index	11 (2–29)	6 (0–21)	0.001
Daily smartphone usage time; hour; median (min–max)	5 (2–10)	4.25 (1–13)	0.069
Duration of smartphone use, years; median (min–max)	10 (6–17)	12 (6–25)	0.082
Age at the onset of smartphone use; median (min–max)	16 (11–34)	19 (11–27)	0.217
Use of smartphone for business; positive, n (%)	30 (81.08%)	34 (89.47%)	0.483
Use of smartphone for social media; positive, n (%)	36 (97.3%)	33 (86.84%)	0.200
Use of smartphone for gaming; positive, n (%)	9 (24.32%)	7 (18.42%)	0.732
Use of smartphone for viewing videos; positive, n (%)	27 (72.97%)	26 (68.42%)	0.858

Kg: kilogram; max: maximum; min: minimum; mm: millimeter; n: number; SD: standard deviation; BMI: body mass index.

**Table 2:** Comparison of neck extensor muscle thickness in patients with and without smartphone addiction.

Parameters	Smartphone addicted (n=37, 49.3%)	Non-addicted (n=38, 50.7%)	p-value
Dominant trapezius; mm, mean (SD)	1.96 (0.64)	1.95 (0.52)	0.944
Dominant splenius capitis; mm, median (min–max)	2.3 (1.1–4.3)	2.3 (1.5–4.3)	0.907
Dominant semispinalis capitis; mm, median (min–max)	3.3 (1.9–6.2)	3.15 (2.2–5.8)	0.787
Dominant semispinalis cervicis; mm, mean (SD)	5.28 (1.43)	4.87 (1.07)	0.163
Dominant multifidus; mm mean (SD)	13.16 (3.09)	12.78 (2.33)	0.557
Non-dominant trapezius; mm, mean (SD)	1.96 (0.59)	1.88 (0.55)	0.540
Non-dominant splenius capitis; mm, median (min–max)	2.3 (1.6–4.3)	2.4 (1.3–4.3)	0.920
Non-dominant semispinalis capitis; mm, median (min–max)	3.3 (2–5.6)	3.1 (2.3–7.5)	0.932
Non-dominant semispinalis cervicis; mm, mean (SD)	4.92 (1.2)	5.13 (1.41)	0.496
Non-dominant multifidus; mm, mean (SD)	13.59 (2.84)	13.44 (2.43)	0.810
Average trapezius; mm, mean (SD)	1.96 (0.59)	1.91 (0.47)	0.716
Average splenius capitis; mm, median (min–max)	2.3 (1.4–4.2)	2.28 (1.55–4.3)	0.886
Average semispinalis capitis; mm, median (min–max)	3.2 (2.15–5.8)	3.2 (2.35–6.65)	0.824
Average semispinalis cervicis; mm, mean (SD)	5.09 (1.23)	5 (1.14)	0.749
Average multifidus; mm mean (SD)	13.37 (2.92)	13.11 (2.28)	0.668

max: maximum; min: minimum; mm: millimeter; SD: standard deviation.

From the ultrasonographic evaluation, we deduced the following: the duration of smartphone usage was found to be negatively correlated with the thickness of the trapezius muscle on the dominant side (ρ=-0.362, p=0.028). Notably, the age of the onset of smartphone use demonstrated positive correlations with the thickness of the splenius capitis muscle on both the dominant (ρ=0.358, p=0.030) and non-dominant sides (ρ=0.487, p=0.002), the thickness of the semispinalis ­cervicis muscle on the dominant side (r=0.332, p=0.045), and the average thickness of the splenius capitis (ρ=0.442, p=0.006) and semispinalis cervicis muscles (r=0.349, p=0.034) (Table 3).

**Table 3:** The effect of the age of the onset of smartphone use on neck extensor muscle thickness.

			Age of smartphone use onset				p-value
			B	R	R^2^	F	
**Dependent variables** †	Dominant trapezius	n=71ɸ	0.020	0.145	0.021	1.489	0.226
	Dominant splenius capitis*		0.007	0.264	0.070	5.178	**0.026**
	Dominant semispinalis capitis*		0.007	0.266	0.071	5.238	**0.025**
	Dominant semispinalis cervicis		0.070	0.230	0.053	3.863	0.053
	Dominant multifidus		0.147	0.227	0.051	3.743	0.057
	Non-dominant trapezius		0.015	0.109	0.012	0.830	0.365
	Non-dominant splenius capitis*		0.239	0.287	0.082	6.182	**0.015**
	Non-dominant semispinalis capitis*		0.005	0.209	0.044	2.928	0.092
	Non-dominant semispinalis cervicis		0.091	0.287	0.069	6.216	**0.015**
	Non-dominant multifidus		0.132	0.211	0.044	3.207	0.078
	Average trapezius		1.630	0.138	0.019	1.333	0.252
	Average splenius capitis*		0.007	0.300	0.090	6.800	**0.011**
	Average semispinalis capitis*		0.007	0.270	0.059	5.282	**0.025**
	Average semispinalis cervicis		0.081	0.286	0.082	6.159	**0.016**
	Average multifidus		0.139	0.225	0.051	3.686	0.059

* Logarithmic transformation was applied to achieve a normal distribution pattern prior to analysis.

† Linearity assumptions were satisfied for all parameters (deviation from linearity; p>0.05).

ɸ Outliers were identified and removed based on Mahalanobis distance, resulting in an optimal analysis conducted with a sample size of n=71.

Statistically significant values are denoted in bold.

The age of commencing smartphone use was categorized based on variance and homogeneity, and measurement ­values were compared accordingly. The analysis showed statistically significant differences in muscle thickness: dominant semispinalis cervicis (4.62±1.05 mm for ages 11–15 years, 5.01±1.33 mm for ages 16–20 years, and 5.51±1.27 mm for ages >20 years, p=0.047), non-dominant semispinalis cervicis (4.47±0.99 mm, 5.11±1.37 mm, and 5.4±1.36 mm, respectively, p=0.043), average splenius capitis (2.15 mm, 2.18 mm, and 2.5 mm, respectively, p=0.027), and ­average ­semispinalis cervicis (4.55±0.91 mm, 5.04±1.25 mm, and 5.45±1.18 mm, respectively, p=0.026). The >20 years’ age group had the ­highest values, while the 11–15 years’ age group had the lowest. No significant differences were found for other parameters.

Regression analysis for examining the effect of the age of smartphone use onset on neck extensor muscle thickness revealed several significant findings. For the dominant splenius capitis (B=0.007, R=0.264, R²=0.070, F=5.178, p=0.026) and the dominant semispinalis capitis (B=0.007, R=0.266, R²=0.071, F=5.238, p=0.025), a statistically significant relationship was observed after logarithmic transformation. Similarly, statistically significant associations were found for the non-dominant splenius capitis (B=0.239, R=0.287, R²=0.082, F=6.182, p=0.015) and the non-dominant semispinalis cervicis (B=0.091, R=0.287, R²=0.069, F=6.216, p=0.015) muscles. Additionally, the average thickness of the splenius capitis (B=0.007, R=0.300, R²=0.090, F=6.800, p=0.011) and semispinalis capitis (B=0.007, R=0.270, R²=0.059, F=5.282, p=0.025) muscles also showed statistically significant relationships after logarithmic transformation. These results suggest that the age of starting smartphone use significantly influences the thickness of certain neck extensor muscles.

## DISCUSSION

This study aimed to investigate the relationship between smartphone usage and neck extensor muscle thickness using ultrasonographic evaluation. The analysis revealed that participants with smartphone addiction exhibited a higher NDI, although no significant differences in neck extensor muscle thickness were observed between addicted and non-addicted individuals. Notably, significant associations were found between the age at which participants began using smartphones and the thickness of various neck extensor muscles. These results indicate that early exposure to smartphone use may significantly influence the morphology of neck extensor muscles.

Our findings contribute to the growing body of evidence indicating that smartphone usage impacts musculoskeletal health. Previous research has primarily focused on the effects of smartphone use on neck disability and cervical disc degeneration^5,6^. Our study uniquely adds to the literature by providing the morphological evaluations of neck extensor muscles. Specifically, we found that the early onset of smartphone use is associated with a reduced thickness in the splenius capitis and semispinalis cervicis muscles. These findings fill a critical gap in the existing research, which has primarily focused on neck disability and cervical disc degeneration without examining the morphological changes in neck muscles.

When comparing our findings with those of other ­studies, we observed both similarities and differences. For example, Rezasoltani et al.^11^ found that the semispinalis capitis muscle was smaller in female office workers with unilateral chronic non-specific neck pain compared to healthy controls. While our analysis did not show differences in the semispinalis capitis muscle between groups categorized by smartphone addiction or the age of onset of smartphone use, regression analysis indicated that the age of onset of smartphone use affects muscle thickness. This suggests a partial correlation with Rezasoltani et al.'s findings. Similarly, Fernández-de-las-Peñas et al.^12^ reported a reduced cross-sectional area of the cervical multifidus muscle in women with chronic bilateral neck pain compared to controls. A key distinction between our study and theirs is that we included participants regardless of whether or not they experienced neck pain, whereas they did not. Additionally, our measurement method focused on muscle thickness, a more practical approach, whereas Fernández-de-las-Peñas et al. measured the cross-sectional area. Karabaş et al.^13^ demonstrated a decrease in neck extensor muscle thickness in patients with ankylosing spondylitis, a condition known to affect the neck region. In a related study, Kuzu et al.^14^ found that the average sonographic thickness values of the neck extensor muscles in patients with fibromyalgia and chronic neck pain were lower than those in asymptomatic controls. Additionally, studies by Bokaee et al.^15^ and Goodarzi et al.^16^ examined neck extensor muscle thickness in individuals with forward head posture. These studies found no differences in muscle thickness at rest; however, Goodarzi et al. observed less dimensional change in multiple neck extensor muscles during isometric contraction, suggesting that forward head posture may impact these muscles. Although our study did not focus on forward head posture, the relationship between smartphone use and postural disorders is well documented in the literature.

This study has some limitations. The cross-sectional design limits the ability to draw causal inferences. Longitudinal studies are needed to establish the temporal relationship between smartphone use and changes in muscle thickness. Additionally, the sample size, although sufficient for this analysis, may limit the generalizability of the findings to broader populations. Future research should aim to replicate these findings in larger and more diverse cohorts.

## CONCLUSION

Our study highlights the significant impact of smartphone use on neck extensor muscle morphology. Early and prolonged smartphone use is associated with a reduced thickness of certain neck muscles, while starting smartphone use at an older age appears to mitigate these effects. These findings emphasize the need for awareness and preventive measures to address the musculoskeletal health implications of smartphone usage in modern society.

## Data Availability

The datasets generated and/or analyzed during the current study are available from the corresponding author upon reasonable request.
